# Vaccination-Related Applications and Health Care Professionals’ Observed Changes in Human Papillomavirus Vaccine Hesitancy: Cross-Sectional Survey

**DOI:** 10.2196/77778

**Published:** 2026-05-14

**Authors:** Manali Desai, Shreela Sharma, Joel Fokom-Domgue, Robert Yu, Wenyaw Chan, Onyema G Chido-Amajuoyi, Charles Darkoh, Sanjay Shete

**Affiliations:** 1 Division of Cancer Prevention and Population Sciences, The University of Texas MD Anderson Cancer Center, Houston, TX, United States; 2 Center for Healthy Communities, Department of Epidemiology, The University of Texas Health Science Center at Houston School of Public Health, Houston, TX, United States; 3 Department of Biostatistics, The University of Texas MD Anderson Cancer Center, 1515 Holcome Blvd, Houston, TX, 77030, United States, 1 2817481063, 1 7137452483; 4 Department of Epidemiology, The University of Texas MD Anderson Cancer Center, Houston, TX, United States; 5 Department of Biostatistics and Data Science, The University of Texas Health Science Center at Houston School of Public Health, Houston, TX, United States; 6 Division of Hematology and Oncology, Department of Medicine, University of Washington, Seattle, WA, United States

**Keywords:** human papillomavirus vaccination, HPV vaccination, vaccine hesitancy, mobile apps or web-based applications, digital health, COVID-19 pandemic, health policy, health care professional, health promotion

## Abstract

**Background:**

Digital tools are known to promote public health interventions such as vaccine delivery. The recommendation that health care professionals (HCPs) use vaccination-related mobile apps or web-based applications has contributed to improving vaccine awareness and acceptance in the United States. The state of Texas, which has one of the lowest human papillomavirus (HPV) vaccination rates, has seen a significant increase in HPV vaccine hesitancy, particularly during the COVID-19 pandemic.

**Objective:**

This study aimed to examine the association between changes in HPV vaccine hesitancy observed by HCPs among patients in Texas and promotion of vaccination-related applications at the health care facilities where they practiced during the COVID-19 pandemic.

**Methods:**

A population-based cross-sectional survey was administered in 2021 by the MD Anderson Cancer Center to HCPs working in Texas using email addresses obtained from the LexisNexis Master Provider Referential Database. HCPs were asked if they assessed HPV vaccination status during every patient encounter. Those who responded “Often/Always” or “Sometimes” were subsequently asked whether they observed any change (“Decreased,” “No change,” “Increased,” or “Not sure”) in HPV vaccine hesitancy during the COVID-19 pandemic. Additionally, HCPs were asked whether their practice offers HPV vaccination. Those who responded “Yes” to this question were further asked whether vaccination-related applications are promoted at the facility where they practice, with response options being “Yes,” “No,” or “I don’t know.” Logistic regression analysis was performed to examine the association between changes in HPV vaccine hesitancy observed by HCPs and promotion of vaccination-related applications at the facility where they practice.

**Results:**

A total of 1283 HCPs completed the survey. Of the 730 HCPs who observed changes in HPV vaccine hesitancy, 51 (7%) reported a decrease in their patients’ HPV vaccine hesitancy. Of these 730 HCPs, 578 (79.2%) responded to the questions regarding vaccination-related applications, of whom 104 (18%) reported that vaccination-related applications were promoted at their facilities. Compared to HCPs who reported not promoting vaccination-related applications, those who reported doing so at their facilities had significantly higher odds of observing a decrease in HPV vaccine hesitancy among patients (adjusted odds ratio [aOR] 2.48, 95% CI 1.10-5.55; *P*=.03). HCPs working at federally qualified health centers or city, county, or public health care facilities (aOR 4.02, 95% CI 1.33-12.14; *P*=.01) and HCPs who administered the HPV vaccine under standing orders at their facilities (aOR 2.91, 95% CI 1.11-7.63; *P*=.03) had significantly higher odds of observing a decrease in HPV vaccine hesitancy at their practices.

**Conclusions:**

Our findings suggest that promoting vaccination-related applications at health care facilities in areas with high HPV vaccine hesitancy such as Texas could further decrease HPV vaccine hesitancy in the population. This may be potentially applicable across diverse health care settings, particularly in the context of pandemic preparedness.

## Introduction

In the United States, approximately 47,984 new cases of human papillomavirus (HPV)–associated cancers are diagnosed annually, including 26,280 cases among women and 21,704 cases among men [1]. Despite the availability of safe and effective HPV prophylactic vaccines that can help prevent these cancers and the adoption of a standardized recommendation schedule in the United States [2-5], HPV vaccination uptake remains suboptimal due to barriers including lack of knowledge, concerns about safety and efficacy, and cost concerns [6,7]. In response, government agencies have made efforts to improve HPV vaccination coverage through school- and community-based vaccination programs, health care professional (HCP)–based interventions, and cost reduction initiatives [8]. Since it was approved in the United States, the HPV vaccine was included in the Vaccines for Children program, which provides free vaccines to uninsured or underinsured children until the age of 18 years [9-11]. Additionally, the Affordable Care Act mandates that private health insurers cover vaccines recommended by the Advisory Committee on Immunization Practices, including HPV, and are prohibited from charging copays or deductibles for these vaccines when administered by in-network HCPs [12]. Despite these efforts, HPV vaccination coverage among adolescents aged 13 to 17 years was only 62.9% in 2024, which is far below the Healthy People 2030 goal of achieving 80% vaccination coverage [7,13,14].

According to the World Health Organization, vaccine hesitancy, one of the major threats to public health, is defined as a delay in accepting or refusal of safe and effective vaccines despite their availability [15,16]. In the context of HPV vaccination, vaccine hesitancy constitutes a significant barrier to both initiating and completing the HPV vaccination series [17]. The most common reasons for vaccine hesitancy are limited knowledge; beliefs that vaccination is unnecessary; concerns about vaccine safety and potential side effects; and a lack of strong, presumptive HCP recommendations [18-23]. A few studies have shown the benefits of using digital interventions to reduce vaccine hesitancy by increasing public awareness and knowledge and intentions to receive the HPV vaccine [24,25]. A quantitative study conducted among 20 adolescents and 34 parents in Philadelphia, Pennsylvania, assessed the effectiveness of the Vaccipack app, which included a vaccine tracker, inspirational stories, and an active discussion forum, designed to promote intention to receive the HPV vaccine [26]. This app, based on an integrated behavioral model, was found to have high acceptability among parents (88%) and adolescents (75%) and to be beneficial for them (82% and 85%, respectively) [26]. Another qualitative study assessing the Vax4HPV mobile app designed based on the social ecological theory was conducted among parents of children and adolescents aged 9 to 14 years in Connecticut to evaluate its benefits in reducing HPV vaccine hesitancy through informed decision-making [27]. This study reported that the implementation of this theory-based, user-centered app helped mitigate hesitancy by bridging the gap between intention to vaccinate and vaccine uptake [27]. Participants reported that the Vax4HPV app provided gender-neutral information, stronger HCP recommendations, and information on clinics providing free vaccination or scheduling appointments [27]. Additionally, a study conducted in New Mexico among 82 parents of vaccine-eligible teenagers found that mobile apps such as Vacteens reduced vaccine hesitancy among parents and their adolescent daughters aged 11 to 14 years [28]. Similarly, a clinic-based study conducted in Houston, Texas, reported that the HPVCancerFree app served as an adjunct during health care visits, providing education and support to help parents make evidence-based decisions about HPV vaccination [29]. A randomized trial among 209 parents and their sons aged 11 to 14 years evaluated the effectiveness of a tailored and engaging mobile app (TeenVac) compared to the Centers for Disease Control and Prevention (CDC) HPV vaccination pamphlet available online [30]. This study showed that using the TeenVac app led to higher completion of the HPV vaccine series than using the pamphlet [30].

Texas is the second-largest state in the United States, with one of the lowest up-to-date HPV vaccination rates (52.2%) across the country [31]. Access to preventive health care during the COVID-19 pandemic was impacted due to closed medical offices, limited availability of appointments, and concerns regarding contracting COVID-19 [32]. A study conducted among 252 parents at a children’s hospital in California reported increased hesitancy regarding childhood vaccines during the COVID-19 pandemic [33]. Furthermore, a survey conducted among HCPs in Texas found that 17.1% of HCPs reported an increase in HPV vaccine hesitancy, whereas another study of pediatricians in Massachusetts found that 11% of pediatricians reported an increase in parental HPV vaccine hesitancy during the pandemic [34,35]. Compared to 2018 and 2019, substantially fewer childhood (9-12 years) and adolescent (13-17 years) vaccines were administered from March 2020 to September 2020 (ie, during the acute phase of the COVID-19 pandemic) [36]. While prior studies have reported the benefits of HPV vaccination–related applications outside major outbreaks or public health emergencies [26-29], little is known about the role that these applications may play during outbreaks such as COVID-19 or other pandemics. In this study, we examined the association between the promotion of vaccination-related applications at health care facilities and changes observed by HCPs regarding HPV vaccine hesitancy during the COVID-19 pandemic. We hypothesized that HCPs who reported promoting vaccination-related applications at their health care facilities had higher odds of observing a decrease in HPV vaccine hesitancy. Grounded in the diffusion of innovation theory, this study’s findings will support the integration of digital health interventions into the package of pandemic preparedness activities in the United States.

## Methods

### Study Design, Data Source, and Population

We conducted a population-based cross-sectional study using data from a statewide survey collected between January 2021 and April 2021 by the University of Texas MD Anderson Cancer Center. This survey was developed to evaluate Texas-based HCPs’ knowledge, perceptions, and practices regarding the HPV vaccine recommendation and was administered electronically in English and Spanish using all email addresses obtained from the LexisNexis Master Provider Referential Database [37]. The survey was sent via REDCap (Research Electronic Data Capture; Vanderbilt University) to Texas-based HCPs practicing family medicine, internal medicine, pediatrics, and gynecology; physician assistants; and nursing staff who held a current Texas license and for whom email addresses were available in the LexisNexis database. Up to 3 email reminders were sent to each participant. In case the survey was not completed after the initial invitation, the first follow-up email was sent on the eighth day (–2 to +2 days) from the initial invitation, the second follow-up email was sent on the eighth day (–2 to +2 days) from the first follow-up email, and the third follow-up email was sent 5 days (–2 to +2 days) after the second follow-up email. The estimated time to complete the survey was 10 minutes. A total of 1283 HCPs completed the survey, with a response rate of 7% (1283/18,328). To report this study, we adhered to the STROBE (Strengthening the Reporting of Observational Studies in Epidemiology) guidelines to ensure ethical and scientific integrity (Checklist 1) [38].

### Ethical Considerations

This study was approved by the University of Texas MD Anderson Cancer Center Ethical Review Board (protocol 2019-1257). Participation in this study was voluntary and confidential. All participants provided informed consent. The dataset underwent deidentification through the removal of all identifiable information. Each participant received a US $10 gift card for completing the survey. Additionally, after completing the survey, participants were entered into a draw for a US $1000 Amazon gift card, which was held 3 months after the survey closed.

### Measures

#### Dependent Variable: Changes in HPV Vaccine Hesitancy Among Patients Observed by HCPs During the COVID-19 Pandemic

We examined the changes in HPV vaccine hesitancy observed by HCPs among patients during the COVID-19 pandemic. To capture the subjective impression of hesitancy, HCPs were asked the following: “At every patient encounter, do you assess HPV vaccination status?” Those who answered “Sometimes” or “Often/Always” were further asked the following: “During the COVID-19 pandemic, I have observed that HPV vaccination hesitancy...” The possible responses were “Decreased,” “Increased,” “No change,” and “Not sure.” We coded “Decreased” as 1 and “Increased or no change” as 0. Those who responded “Not sure” were excluded from this study. The reference category was defined as “Increased or no change.”

#### Independent Variable: Promotion of Vaccination-Related Applications at the HCPs’ Facilities of Practice

For this variable, HCPs were first asked the following: “Does your practice offer HPV vaccination?” Those who answered “Yes, always offered” or “Yes, but not consistently” were assessed for promoting vaccination-related applications at their facilities using the survey question “Which of the following is practiced at your facility? ...Promote vaccination-related apps,” with possible responses of “Yes,” “No,” and “I don’t know.” The reference category was “No.”

#### HCP-Related Variables (Covariates): HCPs’ Sociodemographic Variables and Practice-Related Factors

We included HCPs’ age (<35 years, 35 to 54 years, and ≥55 years), sex (male and female), race and ethnicity (non-Hispanic White, non-Hispanic Black, Asian, Hispanic, and other), location of practice (rural and urban) and type of facility (university hospital, teaching hospital, affiliated clinic, or employed physician practices; Federally Qualified Health Center [FQHC] or city, county, or public health care facility; or other), administration of HPV vaccination under standing orders (yes or no), use of reminders (eg, phone or mail) when HPV vaccination was due (“Yes,” “No,” and “I don’t know”), and use of recalls (eg, computerized tracking) when HPV vaccination was past due (“Yes,” “No,” and “I don’t know”). On the basis of the 2013 Rural-Urban Continuum Codes, we classified the location of practice for the HCPs using the zip codes of their workplaces, with Rural-Urban Continuum Codes 1 to 3 classified as urban and 4 to 9 classified as rural [39].

#### Patient-Related Variables: Patients’ Sociodemographic Factors

As we did not collect data from patients directly, patient-related variables were obtained from the 2022 American Community Survey 5-year database [40], which allowed us to determine the race and ethnicity of the population residing in Texas at the level of the zip code where the HCPs practiced. This variable was also included in our model as a potential predictor of changes in HPV vaccine hesitancy observed by HCPs among their patients [41].

### Data Analysis

Descriptive statistics were used to estimate the prevalence of promoting the use of vaccination-related applications at the HCPs’ facilities and HCP- and practice-level factors stratified by HCPs’ observed changes in HPV vaccine hesitancy during the COVID-19 pandemic. To that end, we used frequencies, weighted percentages, 95% CIs, and the Pearson chi-square test or Fisher exact test as appropriate. Bivariate and multivariable logistic regression analyses were used to determine the odds of observed decrease in HPV vaccine hesitancy reported by HCPs and the promotion of vaccination-related applications during the COVID-19 pandemic. In the multivariable logistic regression model, we adjusted for HCPs’ age, sex, race and ethnicity, practice location, type of facility, administration of HPV vaccination under standing orders, use of reminders (eg, phone or mail) when HPV vaccination was due, use of recalls (eg, computerized tracking) when HPV vaccination was past due, and predominant zip code–level race and ethnicity of patients. Descriptive analyses were conducted using the full sample (n=730), whereas regression analyses were based on a reduced sample (n=576) due to missing data for one or more covariates. However, for each covariate with missing data, a “missing” category was created and included in the logistic regression. We considered *P* values <.05 to be statistically significant, and all tests were 2 sided. We conducted our analysis using SAS (version 9.4; SAS Institute).

## Results

### Demographic Characteristics

Overall, 1283 HCPs completed the survey, with a mean age of 47.1 (SD 11.3) years. Of the 730 HCPs who responded to the survey question about changes in HPV vaccine hesitancy, 51 (7%) reported observing a decrease in HPV vaccine hesitancy, whereas 679 (93%) observed an increase or no change among patients during the COVID-19 pandemic (Table 1). Of the 730 HCPs, 578 (79.2%) responded to the survey question about promoting vaccination-related applications, of whom 104 (18%) reported promoting them at their practices. A higher proportion of HCPs included in this study were aged 35 to 54 years (441/705, 62.6%), female (556/719, 77.3%), non-Hispanic White individuals (369/730, 50.5%), and urban dwellers (699/729, 95.9%). In addition, 50.1% (282/563) used standing orders, 46.3% (274/592) used reminders, and 41% (244/595) used recalls at their facilities to remind eligible individuals to receive the recommended vaccines (Table 1). A higher proportion of HCPs included in this study practiced in zip codes with predominantly non-Hispanic White populations (424/636, 66.7%), followed by those with predominantly Hispanic populations (171/636, 26.9%; Table 1).

The proportion of HCPs who observed a decrease in HPV vaccine hesitancy among patients was higher among those who promoted vaccination-related applications (13/104, 12.5%); those who were aged 55 years or older (15/185, 8.1%), female (41/556, 7.4%), and Hispanic (13/108, 12%); those practicing in rural locations (4/30, 13.3%); those working at FQHCs or city, county, or public health care facilities (15/99, 15.2%); and those using standing orders (23/282, 8.2%) or reminders (18/274, 6.6%; Table 1). A higher proportion of HCPs who observed a decrease in HPV vaccine hesitancy practiced in zip codes with predominantly Hispanic populations (16/171, 9.4%; Table 1).

**Table 1.** Descriptive statistics of the observed changes in human papillomavirus vaccine hesitancy by the health care professionals among patients during the COVID-19 pandemic (n=730)^a^. The responses to the following survey question were excluded from this analysis: “During the COVID-19 pandemic, I have observed that...HPV vaccination hesitancy has...” - “Not sure.”

Characteristic	Total, n/N (%; 95% CI)	Decreased, n/N (%; 95% CI)	Increased or no change, n/N (%; 95% CI)	*P* value^b^
Promoted vaccination-related applications				*.02*
Yes	104/578 (18.0; 14.9‐21.1)	13/104 (12.5; 6.1‐18.9)	91/104 (87.5; 81.1‐93.9)	
No	402/578 (69.6; 65.8‐73.3)	21/402 (5.2; 3.0‐7.4)	381/402 (94.8; 92.6‐97.0)	
I don’t know	72/578 (12.5; 9.8‐15.2)	3/72 (4.2; 0.0‐8.8)	69/72 (95.8; 91.2‐100.0)	
Age (y)				.64
<35	79/705 (11.2; 8.9‐13.5)	6/79 (7.6; 1.7‐13.5)	73/79 (92.4; 86.5‐98.3)	
35-54	441/705 (62.6; 59.0‐66.1)	27/441 (6.1; 3.9‐8.4)	414/441 (93.9; 91.6‐96.1)	
≥55	185/705 (26.2; 23.0‐29.5)	15/185 (8.1; 4.2‐12.1)	170/185 (91.9; 87.9‐95.8)	
Sex				.59
Female	556/719 (77.3; 74.3‐80.4)	41/556 (7.4; 5.2‐9.6)	515/556 (92.6; 90.4‐94.8)	
Male	163/719 (22.7; 19.6‐25.7)	10/163 (6.1; 2.4‐9.8)	153/163 (93.9; 90.2‐97.6)	
Race or ethnicity				.10
Asian	149/730 (20.4; 17.5‐23.3)	12/149 (8.1; 3.7‐12.4)	137/149 (91.9; 87.6‐96.3)	
Hispanic	108/730 (14.8; 12.2‐17.4)	13/108 (12; 5.9‐18.2)	95/108 (88; 81.8‐94.1)	
Non-Hispanic Black	65/730 (8.9; 6.8‐11.0)	4/65 (6.2; 0.3‐12.0)	61/65 (93.8; 88.0‐99.7)	
Non-Hispanic White	369/730 (50.5; 46.9‐54.2)	18/369 (4.9; 2.7‐7.1)	351/369 (95.1; 92.9‐97.3)	
Other^c^	39/730 (5.3; 3.7‐7.0)	4/39 (10.3; 0.7‐19.8)	35/39 (89.7; 80.2‐99.3)	
Location of practice				.17
Rural	30/729 (4.1; 2.7‐5.6)	4/30 (13.3; 1.1‐25.5)	26/30 (86.7; 74.5‐98.9)	
Urban	699/729 (95.9; 94.4‐97.3)	47/699 (6.7; 4.9‐8.6)	652/699 (93.3; 91.4‐95.1)	
Type of facility				*.003*
University hospital, teaching hospital, affiliated clinic, or employed physician practice	197/730 (27; 23.8‐30.2)	10/197 (5.1; 2.0‐8.1)	187/197 (94.9; 91.9-98.0)	
FQHC^d^ or city, county, or public health care facility	99/730 (13.6; 11.1-16.1)	15/99 (15.2; 8.1-22.2)	84/99 (84.8; 77.8-91.9)	
Other^e^	434/730 (59.5; 55.9‐63.0)	26/434 (6.0; 3.8‐8.2)	408/434 (94; 91.8‐96.2)	
Standing orders				*.01*
Yes	282/563 (50.1; 45.9-54.2)	23/282 (8.2; 5.0-11.4)	259/282 (91.8; 88.6‐95.0)	
No	281/563 (49.9; 45.8‐54.1)	9/281 (3.2; 1.1‐5.3)	272/281 (96.8; 94.7‐98.9)	
Reminders (eg, phone or mail)				.94
Yes	274/592 (46.3; 42.3-50.3)	18/274 (6.6; 3.6‐9.5	256/274 (93.4; 90.5‐96.4)	
No	257/592 (43.4; 39.4‐47.4)	15/257 (5.8; 3.0‐8.7)	242/257 (94.2; 91.3-97.0)	
I don’t know	61/592 (10.3; 7.8‐12.8)	4/61 (6.6; 0.3‐12.8)	57/61 (93.4; 87.2‐99.7)	
Recalls (eg, computerized tracking)				.97
Yes	244/595 (41; 37.0‐45.0)	15/244 (6.1; 3.1‐9.2)	229/244 (93.9; 90.8‐96.9)	
No	263/595 (44.2; 40.2‐48.2)	16/263 (6.1; 3.2‐9.0)	247/263 (93.9; 91.0‐96.8)	
I don’t know	88/595 (14.8; 11.9‐17.7)	6/88 (6.8; 1.5‐12.1)	82/88 (93.2; 87.9‐98.5)	
Predominant non-Hispanic White population zip code				.27
Yes	424/636 (66.7; 63.0‐70.3)	26/424 (6.1; 3.8‐8.4)	398/424 (93.9; 91.6‐96.2)	
No	212/636 (33.3; 29.7‐37.0)	18/212 (8.5; 4.7‐12.3)	194/212 (91.5; 87.7‐95.3)	
Predominant non-Hispanic Black population zip code				.74
Yes	36/636 (5.7; 3.9‐7.5)	2/36 (5.6; 0.0‐13.1)	34/36 (94.4; 86.9-100.0)	
No	600/636 (94.3; 92.5‐96.1)	42/600 (7; 5.0‐9.0)	558/600 (93.0; 91.0‐95.0)	
Predominant Hispanic population zip code				.14
Yes	171/636 (26.9; 23.4‐30.3)	16/171 (9.4; 5.0‐13.7)	155/171 (90.6; 86.3‐95.0)	
No	465/636 (73.1; 69.7‐76.6)	28/465 (6; 3.9‐8.2)	437/465 (94; 91.8‐96.1)	

a Missing observations: 152 for promoted vaccination-related applications, 25 for HCP’s age, 11 for provider’s sex, 1 for location of practice, 167 for standing orders, 138 for reminders, 135 for recalls, 94 each for predominant non-Hispanic White, non-Hispanic Black and Hispanic populations ZIP Code.

b *P* values obtained from the chi-square test; *P* values significant at .05 are in italic formatting.

c “Other” for race and ethnicity includes American Indian and Alaska Native and others.

d FQHC: Federally Qualified Health Center.

e “Other” for type of facility includes group practice, solo practice, faith-based hospital or clinic, and others.

### Relationship Between Promotion of the Use of Vaccination-Related Applications at HCPs’ Facilities and Observing a Decrease in HPV Vaccine Hesitancy Among Patients

Compared to HCPs who did not report promotion of vaccination-related applications at their facilities, those who did had significantly higher odds of observing a decrease in HPV vaccine hesitancy among patients during the COVID-19 pandemic (adjusted odds ratio [aOR] 2.48, 95% CI 1.10-5.55; *P*=.03; Table 2). Additionally, compared to HCPs working at university hospitals, teaching hospitals, affiliated clinics, or employed physician practices, HCPs working at FQHCs or city, county, or public health care facilities had significantly higher odds of observing a decrease in HPV vaccine hesitancy among patients during the COVID-19 pandemic (aOR 4.02, 95% CI 1.33-12.14; *P*=.01; Table 2). Compared to HCPs who did not administer the HPV vaccine under standing orders at their facilities, those who did had significantly higher odds of observing a decrease in HPV vaccine hesitancy among patients (aOR 2.91, 95% CI 1.11-7.63; *P*=.03; Table 2).

We did not find a statistically significant association between observed decrease in HPV vaccine hesitancy during the COVID-19 pandemic reported by HCPs and HCPs’ demographic characteristics, such as age, sex, race and ethnicity, location of practice, and use of reminders or recalls at their facilities, as well as patient-related characteristics, such as zip codes with predominantly non-Hispanic White, non-Hispanic Black, and Hispanic populations in which the HCPs practiced (Table 2).

**Table 2.** Logistic regression analyses of the association between the observed changes in human papillomavirus vaccine hesitancy among patients and promotion of vaccination-related applications during the COVID-19 pandemic.

Characteristic	Crude odds ratio (95% CI)	*P* value	Adjusted odds ratio (95% CI)	*P* value
Promoted vaccination-related applications				
No	Reference	Reference	Reference	Reference
Yes	2.59 (1.25‐5.40)	*.01*	2.48 (1.10‐5.55)	*.03*
I don’t know	0.79 (0.23‐2.75)	.71	0.64 (0.17‐2.48)	.52
Age (y)				
<35	Reference	Reference	Reference	Reference
35-54	0.65 (0.23‐1.81)	.41	0.49 (0.16‐1.51)	.21
≥55	1.07 (0.36‐3.15)	.90	0.95 (0.30‐3.08)	.94
Sex				
Female	Reference	Reference	Reference	Reference
Male	0.62 (0.25‐1.51)	.29	0.45 (0.15‐1.38)	.16
Race or ethnicity				
Asian	1.25 (0.50‐3.18)	.63	1.40 (0.51‐3.80)	.51
Hispanic	2.63 (1.16‐5.97)	*.02*	2.12 (0.77‐5.84)	.15
Non-Hispanic Black	1.18 (0.33‐4.28)	.80	0.82 (0.13‐5.31)	.83
Non-Hispanic White	Reference	Reference	Reference	Reference
Other^a^	1.38 (0.30‐6.41)	.68	3.11 (0.47‐20.42)	.24
Location of practice				
Rural	Reference	Reference	Reference	Reference
Urban	0.53 (0.15‐1.85)	.32	0.57 (0.13‐2.43)	.45
Type of facility				
University hospital, teaching hospital, affiliated clinic, or employed physician practice	Reference	Reference	Reference	Reference
FQHC^b^ or city, county, or public health care facility	4.45 (1.74‐11.42)	*.002*	4.02 (1.33‐12.14)	*.01*
Other^c^	1.15 (0.46‐2.86)	.77	0.90 (0.29‐2.81)	.85
Standing orders				
No	Reference	Reference	Reference	Reference
Yes	2.71 (1.23‐5.98)	*.01*	2.91 (1.11‐7.63)	*.03*
Reminders (eg, phone or mail)				
No	Reference	Reference	Reference	Reference
Yes	1.15 (0.57‐2.35)	.70	0.82 (0.29‐2.33)	.71
I don’t know	1.14 (0.36‐3.6)	.82	0.68 (0.13‐3.60)	.65
Recalls (eg, computerized tracking)				
No	Reference	Reference	Reference	Reference
Yes	1.04 (0.5‐2.16)	.92	0.73 (0.27‐2.00)	.54
I don’t know	1.13 (0.43‐3.0)	.81	1.03 (0.26‐4.07)	.97
Predominant non-Hispanic White population zip code				
No	Reference	Reference	Reference	Reference
Yes	0.55 (0.27‐1.13)	.10	0.81 (0.27‐2.42)	.71
Predominant non-Hispanic Black population zip code				
No	Reference	Reference	Reference	Reference
Yes	1.04 (0.24‐4.62)	.96	0.88 (0.26‐3.03)	.84
Predominant Hispanic population zip code				
No	Reference	Reference	Reference	Reference
Yes	1.94 (0.93‐4.05)	.08	0.81 (0.29‐2.24)	.68

a “Other” for race and ethnicity included American Indian and Alaska Native and others.

b FQHC: Federally Qualified Health Center.

c “Other” for type of facility included group practice, solo practice, faith-based hospital or clinic, and others.

## Discussion

### Principal Findings

In this population-based study, we found that HCPs working in Texas who promoted vaccination-related applications at their facilities had higher odds of observing a decrease in HPV vaccine hesitancy among patients during the COVID-19 pandemic. Our study findings align with those of previous studies reporting the benefits of vaccination-related mobile apps in decreasing HPV vaccine hesitancy and thereby increasing vaccine uptake [24-29]. Recent studies have reported a rise in HPV vaccine hesitancy and decreased vaccine uptake following the COVID-19 pandemic in the United States [33-35,42]. As the HPV vaccine is safe and effective in preventing HPV-associated cancers [2,5], and considering that digital interventions have been proven effective at increasing HPV vaccine awareness and knowledge [24,25], there is a need to promote vaccination-related applications to increase awareness and knowledge and address misinformation about the HPV vaccine.

Previous studies have reported that the most common reasons for HPV vaccine hesitancy are safety concerns and lack of knowledge and absence of HCP recommendation among parents of vaccine-eligible children and among age-eligible adults, respectively [43,44]. A positive association was found between HCPs’ knowledge of the HPV vaccine and initiation and completion of the vaccine series [45]. When HCPs are well informed, they can confidently communicate vaccine benefits and respond to parental concerns, which is critical for building trust and influencing vaccination decisions [46]. Mobile apps have helped users access immunization-related information, create and track vaccination schedules, and locate clinics where the public can receive vaccines [47,48]. Prior studies have focused on using mobile apps to promote HPV vaccination–related information, some of which are publicly available whereas others are still being evaluated as part of research projects. Among the mobile apps under evaluation in research studies, Vaccipack, Vacteens, Vax4HPV, HPVCancerFree, and TeenVac have demonstrated their potential to decrease HPV vaccine hesitancy [26-30]. Thus, integrating vaccination-related mobile apps into clinical practice could be a valuable tool for promoting HPV vaccination and reducing hesitancy. Furthermore, timely reminders sent by mobile apps to vaccine-eligible individuals about their vaccination dates align with the transtheoretical model of behavior change, which posits that behavior change is a gradual process that occurs in stages [49]. By providing reminders to vaccine-eligible individuals, these vaccination-related apps motivate them to move from contemplation to action, thereby improving HPV vaccination uptake [49]. The US CDC developed a publicly available app called CDC Vaccine Schedules to provide HCPs and patients with immediate access to comprehensive information on vaccine recommendations, including immunization schedules, precautions, and contraindications [50]. Other publicly available immunization-related apps include ACOG, developed by the American College of Obstetricians and Gynecologists; HPV Vaccine: Same Way, Same Day, developed by the Academic Pediatric Association and American Academy of Pediatrics; and Vaccines on the Go: What You Should Know [51]. Despite the variety of official vaccination-related applications available to HCPs, we found that a small proportion (104/578, 18%) of Texas-based HCPs promoted HPV vaccination–related applications at their health care facilities.

We also found that HCPs working at FQHCs or city, county, or public health care facilities had higher odds of observing a decrease in HPV vaccine hesitancy among patients than those working at university hospitals, teaching hospitals, affiliated clinics, or employed physician practices. This could be attributed to the greater availability of preventive health care services, such as HPV vaccination, at public facilities, which provides an opportunity for HCPs working at such practices to further counsel patients and recommend vaccination applications [52,53]. A study conducted among HCPs serving minority and low-income populations identified a lack of preventive care visits as a barrier to HPV vaccine recommendation [54]. Given that FQHCs primarily offer health care services to uninsured and low-income patients, they play a critical role in increasing the HPV vaccination uptake among socioeconomically disadvantaged populations [55]. Adolescents from more educated and higher-income households show higher rates of HPV vaccine hesitancy [56]. On the other hand, adolescents with lower income levels have higher odds of completing the HPV vaccine series [57]. These findings highlight the need for organizational policies or protocols to strengthen access to HPV vaccination, improve patient counseling, and reduce vaccine hesitancy.

We found that HCPs who reported using standing orders for HPV vaccination at their facilities had higher odds of observing a decrease in HPV vaccine hesitancy among their patients. Standing orders authorize nurses, pharmacists, and other trained HCPs, as permitted by state law, to assess a patient’s immunization status and administer vaccines in accordance with a preapproved protocol established by the medical director, physician, or other authorized practitioner [58]. A 2022 study conducted among 16 nurses, medical assistants, and HCPs working in primary care settings in the United States found that implementing standing orders for HPV vaccination increased nurses’ and medical assistants’ autonomy [59]. This approach led to improved access to the HPV vaccine, fewer missed opportunities, earlier vaccination, and normalization of HPV vaccination as a routine part of care [59]. As standing orders have been shown to be effective at increasing vaccination rates [60], they may be particularly effective in addressing HPV vaccine hesitancy by ensuring timely, consistent, and standardized HPV vaccination practices across health care facilities.

### Strengths and Limitations

The major strength of our study is the inclusion of HCPs from diverse backgrounds and specialties. While it offers critical insights into the benefits of digital interventions during the COVID-19 pandemic, our study is not without limitations. First, we could not infer causality because the study was cross-sectional. Second, a small proportion of HCPs practicing at the health care facilities who responded to the survey observed decreases in HPV vaccine hesitancy, which may have reduced the power of our statistical analysis. This small sample size also limits the generalizability of our findings and increases the risk of falsely rejecting our study hypothesis. Third, Texas has a unique sociopolitical landscape regarding vaccine attitudes, as well as a distinct population racial, ethnic, and age distribution, limiting the generalizability of our findings to other US regions. Fourth, there is potential for information bias as changes in HPV vaccine hesitancy were self-reported and based on HCP recall. Fifth, the study question does not allow us to specify the types of applications being promoted or the mode of promotion: active (eg, discussed with patients), passive (eg, posters), or both. Sixth, we did not include HCPs comfort with digital tools, patients’ socioeconomic status, and institutional culture as covariates in our study as such information was not available. Finally, the sampling frame derived from the LexisNexis database may not fully cover all Texas-based HCPs as some HCPs may have been missing from the database or lacked an email address, potentially limiting the generalizability of the findings.

### Conclusions

This study demonstrated the added value of mobile apps in reducing HPV vaccine hesitancy in Texas. Our findings have implications for designing and implementing tailored digital interventions to successfully counter misinformation about HPV vaccination, especially during major outbreaks such as the COVID-19 pandemic. Future prospective studies could explore the long-term impact of these vaccination-related mobile apps in reducing missed opportunities and increasing HPV vaccination uptake among age-eligible individuals.

## Supplementary material

10.2196/77778 Checklist 1 STROBE checklist.

## Abbreviations

aOR: adjusted odds ratio
CDC: Centers for Disease Control and Prevention
FQHC: Federally Qualified Health Center
HCP: health care professional
HPV: human papillomavirus
REDCap: Research Electronic Data Capture
STROBE: Strengthening the Reporting of Observational Studies in Epidemiology
